# The Australian–Indonesian monsoon through time

**DOI:** 10.1093/nsr/nwag473

**Published:** 2026-08-05

**Authors:** Mahyar Mohtadi, Martina Hollstein, Mehdi Pasha Karami, Haowen Dang, Zhimin Jian, Andreas Lückge, James M Russell, Stephan Steinke

**Affiliations:** MARUM-Center for Marine Environmental Sciences, University of Bremen, Bremen 28359, Germany; Faculty of Geosciences, University of Bremen, Bremen 28359, Germany; MARUM-Center for Marine Environmental Sciences, University of Bremen, Bremen 28359, Germany; Rossby Centre, Swedish Meteorological and Hydrological Institute, Norrkoping SE-601 76, Sweden; State Key Laboratory of Marine Geology, Tongji University, Shanghai 200092, China; State Key Laboratory of Marine Geology, Tongji University, Shanghai 200092, China; Federal Institute for Geosciences and Natural Resources, Hannover 30655, Germany; Department of Earth, Environmental, & Planetary Sciences, Brown University, Providence, RI 02912, USA; Department of Geological Oceanography, College of Ocean and Earth Sciences, Xiamen University, Xiamen 361102, China; State Key Laboratory of Marine Environmental Science, College of Ocean and Earth Sciences, Xiamen University, Xiamen 361102, China

**Keywords:** monsoon, rainfall, orbital forcing, sea level, Indo-Pacific Warm Pool, AMOC, LGM, mid-Holocene

## Abstract

The Australian–Indonesian monsoon (AIM) is the manifestation of seasonal rainfall change and wind reversal over northern Australia and the Maritime Continent, within a region of extreme land-sea fragmentation. Past AIM variability and change have been related to large, covarying shifts in the boundary conditions such as the atmospheric CO_2_, Northern Hemisphere ice sheets, orbital insolation, and sea-level variations. This review synthesizes what is currently known about AIM evolution and mechanisms, emphasizing the Quaternary where proxy evidence, dating control, and modeling experiments are most abundant. We assess how model hierarchies have been used to separate rainfall and circulation responses, diagnose the role of SST pattern changes and inter-hemispheric energy balance, and show whether shelf exposure should be treated as a first-order monsoon control or a modest boundary condition adjustment. We argue that progress now hinges more on explicit quantification of data uncertainty and proxy-aware evaluation, as well as using isotope-enabled transient simulations and proxy system models that compare models to what proxies record rather than to precipitation alone.

## INTRODUCTION

The Australian–Indonesian monsoon (AIM) appears as the austral summer intensification of rainfall and the austral winter dry southeasterly trade winds over northern Australia and parts of the Maritime Continent (Fig. 1, Fig. S1). The AIM occupies a unique position within the global monsoon system. It spans the equatorial region and is organized not only by large-scale ocean and atmosphere circulations but also by an unusually complex and variable geography that exerts a strong control on surface fluxes and convection [1]. The absence of strong topographic forcing and the presence of a direct near-field ocean results in a spatially structured AIM climate [2], particularly between the equatorial Indo-Pacific Warm Pool (IPWP) and the strictly monsoonal southern Maritime Continent and northern Australia [3]. To understand the AIM, it is more useful to define it as a coupled system in which atmospheric convection over the Maritime Continent, northern Australian hydroclimate, and IPWP temperatures are mutually interacting elements. It is therefore not surprising that the AIM realm remains one of the hardest places for climate models to simulate, especially with respect to the spatial distribution of rainfall, the diurnal cycle, and cloud-convection coupling [4].

**Figure 1.**
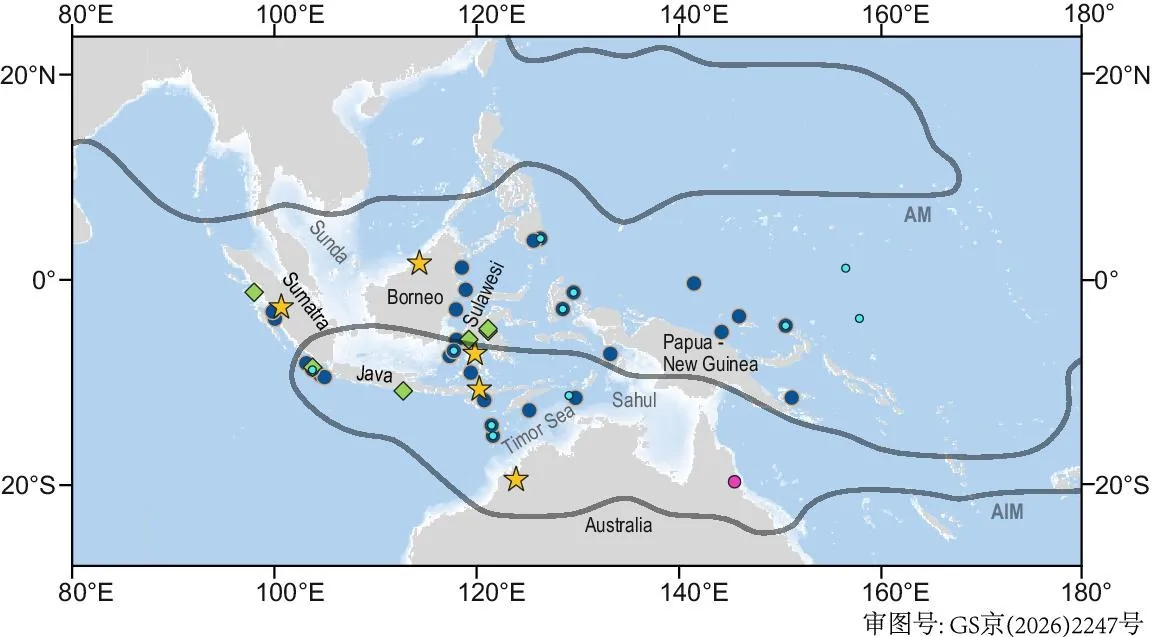
Location of the AIM proxy records. Map of the locations of seawater δ^18^O (blue and cyan dots) and δD records (green diamonds) from lakes and marine sediments, and δ^18^O records of cave speleothems (yellow stars) shown in Fig. 3. Marine records indicated by cyan color are additionally shown in Fig. 2. The magenta dot denotes the location of Lynch’s Crater deposits. The thick gray lines indicate the boundaries of the AIM and the Asian monsoons (AM) according to ref. 102, light blue–white areas show the continental shelf regions. For references and sources, see Table S1.

Unlike many monsoon regions where coastline changes affect the convective core only marginally, the convective engine of the AIM lies directly over the region home to the largest continental shelf regions at low latitudes, the Sunda and Sahul shelves (Fig. 1). Therefore, sea level change may alter the convection, the distribution of evaporation sources, and the near-surface fluxes that influence the seasonal cycle. Glacial-interglacial sea level changes on the order of ∼100 m during the Quaternary repeatedly exposed and flooded the Sunda and Sahul shelves, shifting coastlines by hundreds of kilometers and transforming shallow seas into land and vice versa, providing an ideal testbed to understand the feedback mechanisms to this changing boundary condition [5]. However, because shelf exposure and inundation modify precisely those aspects of geography that organize regional convection, climate model biases can translate directly into biased sensitivity of AIM response. In addition, CO_2_ changes [6], the waxing and waning of Northern Hemisphere ice sheets [7,8], and orbital insolation variations [9,10] shaped the global energy balance on the same timescales as sea level variations, and thus the AIM during the Quaternary. AIM evolution thus presents a twofold scientific challenge: the classic problem of separating covarying forcing mechanisms [11], and a regional modeling problem [12].

This review is organized around mechanisms rather than chronologies, and focuses on the Quaternary where proxy coverage, chronological control, and model experiments are richest. Throughout, we use proxies to motivate hypotheses and constrain spatiotemporal patterns, while models are used for separating forcings and pathways, and translating boundary conditions into physically testable mechanisms.

## THE MODERN AIM: A BASELINE TEST

Like other monsoon systems, the thermal contrast due to differential heating between land and sea causes a reversal in lower tropospheric winds and acts as a seasonal preconditioning for the onset of the AIM [1,13]. During austral summer from December to March, northwesterly and westerly winds transport moist air toward the AIM domain and cause coastal downwelling, high sea surface temperature (SST) of >28°C and a relatively thick mixed-layer in the Indonesian Seas. Together with the arrival of the Intertropical Convergence Zone (ITCZ), these conditions result in deep atmospheric convection and high rainfall in the Australian–Indonesian sector (Fig. S1b). During austral winter when the ITCZ lies in the Northern Hemisphere, dry south-easterly trade winds originating from the high-pressure cell over Australia induce seasonal upwelling, a 2–3°C drop in the SST, and relatively dry conditions over the AIM domain (Fig. S1c). Yet, the AIM is not simply a seasonal migration of the ITCZ. First, the onset of the monsoon is triggered by the arriving Madden–Julian Oscillation and/or the intrusion of a midlatitude trough [13]. Second, rainfall over the Maritime Continent is strongly structured by the diurnal cycle and mesoscale organization [14]. Convection tends to peak over land in the afternoon and propagate offshore at night, with island-scale circulations and strong intraseasonal variability shaping rainfall maxima and moisture recycling [1,14]. In addition, Indo-Pacific gradients in SST and thermocline depth, and the associated changes in Walker circulation (Fig. S1a) that give rise to climate phenomena called El Niño-Southern Oscillation (ENSO) and the Indian Ocean Dipole (IOD), strongly affect the AIM strength on interannual to decadal timescales [15].

Because the AIM depends on the spatial structure of convection (e.g. [16,17]), the AIM cannot be assessed solely by large-scale circulation indices. Many global models show persistent biases over the Maritime Continent and northern Australia, including displaced precipitation maxima, erroneous cloud radiative effects, and a diurnal cycle that is too weak or incorrectly phased [4,18,19]. These biases matter for past simulations because a model that produces too much rain over adjacent seas and too little over islands [12] will respond differently to coastline migration than a model with a realistic land-sea rainfall contrast. Simulations of the past AIM behavior are therefore more reliable when process-relevant benchmarks—seasonal cycle timing, land-sea contrast in rainfall, diurnal amplitude and phase, and moisture convergence patterns—are included rather than relying only on broad monsoon indices.

## PRE-QUATERNARY STUDIES OF AIM EVOLUTION

The pre-Quaternary evolution of the AIM remains poorly constrained owing to the scarcity of long-term paleoclimate archives. Early interpretations based on paleontological and paleosol evidence from central and northern Australia proposed that monsoonal conditions emerged during the Late Eocene to Early Oligocene (∼40–30 Ma). Collectively, these studies suggested that strong seasonal rainfall regimes were already established shortly after Australia’s separation from Antarctica [20]. However, recent reassessments have questioned the evidence for an Eocene–Oligocene Australian monsoon, highlighting substantial uncertainties in the age, preservation, and taxonomic interpretation of the available floral records [21].

Climate model simulations further complicate the early monsoon hypothesis. Eocene simulations indicate that Australia was positioned too far south to experience direct influence from the ITCZ, and that rainfall seasonality differed substantially from the modern monsoon system [22]. Likewise, Miocene simulations suggest that northern Australia experienced monsoonal circulation that was considerably weaker than today [23]. A recent review evaluating tectonic reconstructions, climate model simulations, and marine sedimentary records suggests that near-modern boundary conditions for the AIM developed only after ∼10 Ma, when the northern margin of the Australian Plate migrated north of ∼30°S [21]. The establishment of a fully monsoon-like (‘pseudo-monsoon’) regime took place after ∼3.5 Ma during the Pliocene [21].

## QUATERNARY RECONSTRUCTIONS OF AIM EVOLUTION

The Quaternary contains several testbeds that have shaped what we know about AIM mechanisms. For example, during the Last Glacial Maximum (LGM) between 19 000 and 23 000 years ago (19–23 ka), CO_2_, ice sheets, and sea level differed markedly from today. The LGM is therefore a central benchmark for testing whether models reproduce the broad direction and spatial pattern of hydroclimate anomalies inferred from proxies. Deglaciations add a transient dimension, during which sea level rises rapidly, CO_2_ increases, and ice sheets retreat on millennial timescales. This provides a way to test not only whether models reproduce differences between two equilibria, that is, glacial versus interglacial conditions, but whether they reproduce the timing and sequence of hydroclimate shifts. Although transient simulations remain limited and interpreting proxy records can be complex, deglacial trajectories are a powerful opportunity for model-data integration because they combine strong forcing with robust feedbacks. Finally, interglacial periods provide useful information on insolation forcing since other forcings such as ice sheet and CO_2_ are negligible. Interglacial records are therefore frequently used to test hypotheses about seasonality changes and the response to orbital precession [24,25].

The Quaternary AIM proxy record includes speleothems from parts of Indonesia and Australia, marine sediments from the Indonesian seas and adjacent basins, and terrestrial and nearshore indicators of runoff, vegetation, and sediment transport (e.g. [26]). For the late Quaternary, coral archives add constraints on surface ocean state and hydroclimate variability [27]. A central issue is that proxies record rainfall changes indirectly and qualitatively. They might also reflect processes other than changes in the rainfall amount. For example, rainfall isotopes (δ^18^O and δD) can reflect the seasonality of rainfall, shifts in moisture source between ocean basins, and changes in temperature or upstream rainout history [28–30].

One important note is that monsoon winds and rainfall need not vary in phase, as low-level winds respond primarily to hemispheric pressure gradients and temperature contrasts, whereas rainfall depends on moisture availability and the efficiency of deep convection. Data from a marine sediment core offshore southern Java shows a pronounced decoupling between coastal upwelling (a proxy for winter AIM winds) and terrigenous runoff (a proxy for summer AIM rainfall) since the last glacial period [31]: while AIM winds strengthened over the deglaciation and early Holocene, AIM rainfall changed only on millennial scale without any orbital-scale trend. This asymmetry demonstrates that changes in monsoon rainfall cannot be inferred directly from changes in circulation, and that winds and rainfall respond to different forcing mechanisms at different seasons. This marine evidence provides a crucial framework for interpreting terrestrial records that show glacial aridity despite indications of persistent or intensified circulation, such as leaf wax data from Lake Towuti in Sulawesi [8,28].

Despite difficulties in interpreting the proxy records, several broad points are robust in the Quaternary. Hydroclimate across the Australian–Indonesian sector changes substantially between glacial and interglacial states, implying sensitivity to the combination of CO_2_, ice sheets, and sea level. Many records show strong orbital pacing, consistent with insolation control on seasonal hydroclimate, though the phase and amplitude of response can differ among sites and proxies [32]. The heterogeneity among different subregions of Indonesia and northern Australia is consistent with a system in which both remote forcing and local geography shape climate variability.

### Orbital pacing: precession, obliquity, and insolation gradient

Orbital precession and the tilt of the rotational axis of the Earth (obliquity) modify the incoming solar radiation (insolation) and are therefore expected to influence monsoon seasonality and strength [33]. Because the Maritime Continent anchors convection geographically, insolation-driven changes can shift the atmospheric convection center and alter the seasonal migration of rainfall. Ocean coupling can mediate or amplify these effects by shaping SST gradients within the Indo-Pacific realm.

At orbital timescales, the AIM exhibits strong precession- and obliquity-paced variability. A precession forcing of monsoon rainfall and wind through changes in the state of ENSO has been proposed for the Holocene [34] and over several glacial-interglacial cycles [35,36]. Over the past 400 ka, precession minima and obliquity maxima appear to cause rainfall increase over northwest Australia [37] through expansion and/or latitudinal migration of the ITCZ, respectively [38]. Australian interior lake records provide evidence for orbital-scale modulation of monsoon penetration into arid Australia [9]. Likewise, seawater oxygen isotope (δ^18^Osw) data—a proxy for past changes in ocean salinity—show a dominant precession pacing offshore northern Papua New Guinea over the last glacial-interglacial cycle [39]. Obliquity and precession have been shown to control rainfall over southeast Papua New Guinea during the past 300 ka [40].

The SST and thermocline temperature records from the IPWP follow precessional cycle during the past 25 ka, with higher temperatures during precession minimum [32]. Dynamically, these studies suggest that precession forcing modulates rainfall by affecting ENSO [32,34,39]. This means that the expression of precession forcing is often through changes in zonal temperature gradients, vertical ocean structure, and the continental reach of monsoon rainfall rather than through uniform changes in annual mean precipitation [32]. Another compilation study also suggests a precessional forcing of the upper ocean heat content and salinity in the IPWP and landward moisture transport during the past 360 ka, but more directly without involving ENSO (Fig. 2a and b) [41].

**Figure 2.**
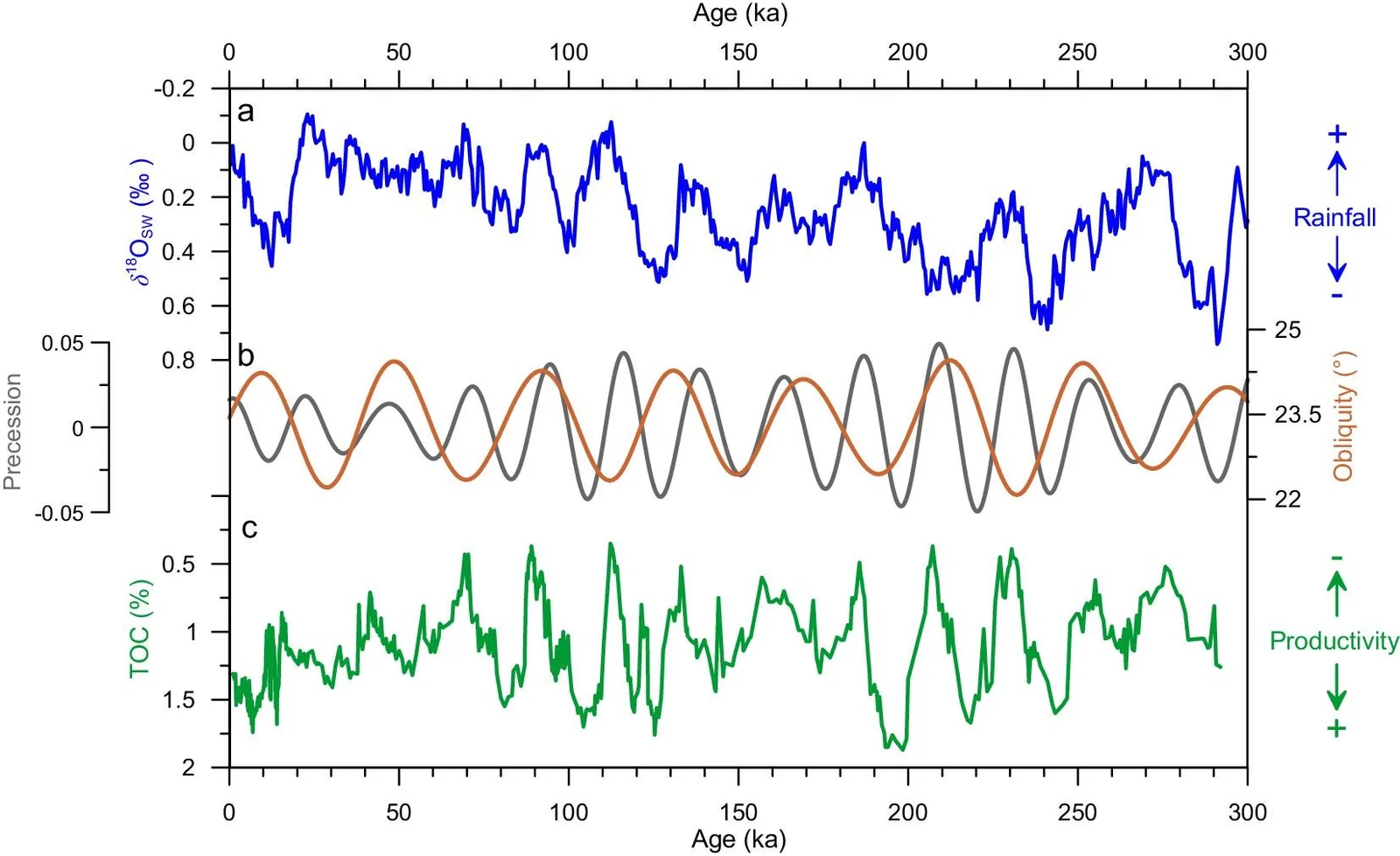
Orbital changes in reconstructed AIM rainfall and wind over the past 300 ka. (a) Seawater δ^18^O of the IPWP records (blue) indicating rainfall-driven changes in ocean salinity [41]. (b) Precession (gray) and obliquity (orange) indices [103]. (c) Total organic carbon (TOC, green, inverted axis) content in marine sediments offshore southwest Sumatra, indicating AIM wind-related changes in upwelling intensity [43]. For locations, see cyan dots in Fig. 1. For spectral power, the reader is referred to the original publications.

In the eastern Indian Ocean, proxies for continental runoff also show precessional cyclicity over several glacial–interglacial cycles [42,43], suggesting a dominant control of precession on AIM rainfall. There, thermocline temperature and marine productivity changes linked to upwelling also show a clear precession pacing, offshore southern Sumatra [43,44], southern Java [31], as well as in the Timor Sea [45]. Northwest Australian dust records also vary at obliquity band and with Summer Inter-Tropical Insolation Gradient (SITIG, 23°N–23°S) [46] over the past three million years [47]. SITIG itself is controlled by precession and obliquity and drives Hadley circulation strength and cross-equatorial moisture transport, and ultimately, monsoon strength [48]. These wind proxy data suggest that precession and obliquity also control monsoon wind strength on orbital scales (Fig. 2b and c).

### Sea-level changes: shelf exposure and inundation

On glacial–interglacial timescales, proxy evidence converges on land–sea geometry as a first-order control on AIM rainfall. Exposure of the Sunda and Sahul shelves during glacial lowstands dramatically altered the surface energy and moisture budgets of the Maritime Continent. Pollen, geomorphological, and biogeographic evidence indicates widespread drying and increased rainfall seasonality across central Sundaland, accompanied by expansion of savanna and open woodland [26,49]. Several terrestrial records demonstrate that this drying occurred even when monsoon circulation remained active. Lake Towuti records show pronounced glacial aridity in central Sulawesi coeval with evidence for intensified monsoon circulation [8,28]. Glacial vegetation across eastern Java shows a strong spatial heterogeneity, with ecosystems responding differently depending on altitude and distance to shelf areas [50].

The mechanisms by which shelf exposure is proposed to influence precipitation include evaporation limitation [28], changes in moisture trajectory [51], or displacement of convection center [52,53]. Shelf exposure reduces the oceanic surface area available for evaporation, increases land fraction and sensible heating, and lowers boundary-layer moist static energy [54]. Soil and vegetation feedbacks amplify this effect: nutrient-poor sandy shelf sediments, combined with low atmospheric CO_2_ and increased seasonality, favor grasses and savanna vegetation [49,50,55], while oceanic feedbacks further cool the atmosphere and contribute to atmospheric cooling during glacial sea level lowstands [56]. Marine records from the eastern- and westernmost parts of the Maritime Continent indicate that the center of convection might have shifted over the ocean during glacials [52,53]. The development of a discontinuous ‘savanna corridor’ [49] through Sundaland thus reflects a relocation of the convection from the heart of the Maritime Continent toward the adjacent oceans, rather than circulation collapse.

The last deglaciation is central for AIM studies because it combines rapid sea level rise with increasing CO_2_ and retreating ice sheets, and because multiple archives have comparatively robust chronologies. Several speleothem records highlight rapid hydroclimate transitions during this period: cave stalagmites from Flores Island show an intensification of rainfall that has been linked to sea-level rise and shelf inundation rather than to local insolation alone [51], and northern Australian speleothems suggest an abrupt AIM strengthening at the end of the deglaciation, interpreted as evidence for sectoral decoupling and threshold-like emergence of modern monsoon behavior [57]. These findings support a hypothesis particularly relevant to the AIM, that shelf flooding altered the geography of convection and moisture convergence, generating relatively abrupt and spatially heterogeneous responses.

Terrestrial lake and ecosystem records both strengthen and complicate the picture. Mineral composition and δ^13^C of plant waxes in Lake Towuti, Sulawesi, indicate strong glacial aridity and ecosystem reorganization [8]. A deuterium isotope (δD) record of plant waxes from the same lake suggests that intensified monsoon circulation can coincide with local aridity, implying decoupling between circulation strength and effective moisture depending on convergence position, moisture availability, and seasonality [28]. Records from central Indonesia more broadly document hydrology-vegetation shifts since the LGM [58], reinforcing that land-surface responses and ecological thresholds are integral parts of the AIM story. Records of pollen and δ^13^C of plant waxes from a sediment core offshore south Sumbawa Island indicate that the winter AIM was drier during the last glacial compared to the Holocene [55], while paired leaf wax carbon isotopes (δ^13^C) and δD records offshore south Java, just 6° farther to the west, indicate extended dry season but a wetter rainy season, that is, a stronger seasonality of the AIM, during the last glacial [50]. Finally, a compilation study of rainfall proxy records suggests an increase in dry season precipitation along with shelf inundation during the last deglaciation [59]. A pivotal marine record off southern Java separates winter monsoon winds (upwelling indicators) from summer monsoon rainfall/runoff (terrigenous proxies), demonstrating that these two seasonal components can show different deglacial trajectories [31]. This seasonal duality matters because it implies that winds and rainfall are not interchangeable ‘monsoon strength’ metrics, and that different forcing pathways may dominate different seasons.

### Millennial events: interhemispheric see-saw and ITCZ dynamics

Millennial scale fluctuations in the Atlantic Meridional Overturning Circulation (AMOC) strength during glacial periods changed interhemispheric thermal gradient, and are well expressed in numerous monsoon records across the globe [33]. One of the clearest signals during the last deglaciation in the Australian–Indonesian sector is the imprint of Heinrich Stadial 1 (HS1, 14.7–18.2 ka), expressed through reorganizations of rainfall, winds, and the spatial structure of the tropical rain belt. Northwestern Australian speleothem δ^18^O records have been interpreted as indicating wetter conditions during Heinrich stadials and the Younger Dryas (11.7–12.9 ka) and drier conditions during Dansgaard–Oeschger interstadials [57,60]. Wetter conditions during HS1 are also indicated in Lynch’s Crater in northeastern Australia [61], in the southern Maritime Continent—in Flores speleothem δ^18^O [62] and in the Flores Sea sediments [63]—all being interpreted with meridional ITCZ migrations under interhemispheric energy balance changes [64]. In this scenario, a cooler Northern Hemisphere during Heinrich stadials shifts the ITCZ toward the warmer Southern Hemisphere, which transports heat back to the Northern Hemisphere through the upper limb of the Hadley circulation [7]. However, the meridional displacement of the ITCZ was spatially non-uniform, much like the modern ITCZ, and might have been at a more northerly position over NW Australia compared to the regions farther east [65].

Proxy records from Lake Towuti in Sulawesi at ∼4°S suggest a more complex hydroclimate response. Lake-level reconstructions indicate drier conditions during HS1 and HS2 [66], similar to plant-wax isotope records [8], despite evidence for a stronger AIM circulation [28]. Farther north and west, HS1 is predominantly characterized by drier conditions, as inferred from stalagmite δ^18^O records in northern Borneo at ∼5°N [67] and western Sumatra near the equator [68], as well as from seawater δ^18^O and terrigenous components in marine sediment records from the eastern Indian Ocean between 3°N and 8°S [7,31]. Equatorial sites might have been further buffered by competing seasonal moisture sources. Borneo and Sulawesi receive rainfall from boreal and austral monsoon pathways as well as from local oceanic convection, allowing anomalies along one pathway to be partially compensated by another [28]. Consequently, the absence of Younger Dryas or other, weaker Northern Hemisphere abrupt climate events in equatorial IPWP records [36,69–71] might indicate threshold dynamics and convective cancellation, rather than the absence of external forcing. Lastly, we note that these regions lie outside of the AIM domain (see Fig. 1), within the tropical ever wet region that does not fulfill the seasonality criterion for a monsoon realm [33].

A major recent development is the move from site-specific interpretations toward spatially explicit reconstructions of ITCZ variability across the IPWP. An earlier synthesis of paleoenvironmental changes within the AIM realm did not address millennial-scale dynamics during the past 30 ka [26]. A more recent study investigates the spatial pattern of seawater δ^18^O records as a proxy for past ocean salinity and, by inference, rainfall, and suggests a contracted tropical precipitation belt located at about 10°N and dry conditions in the AIM realm during the early HS1 [72]. In contrast, another compilation of rainfall proxy records across the same latitudes indicates wetter conditions north of Australia and drier conditions north of the equator during early HS1 [73]. Empirical orthogonal function of the rainfall-related δ^18^O records shows that the first principal component explains more than 80% of the variance and is related to glacial–interglacial changes in boundary conditions, 12% is explained by meridional shift of the ITCZ, while only 4% of the variance can be explained by contraction or expansion of the ITCZ [74].

Principal component analysis of the available seawater δ^18^O records within or close to the AIM realm proves useful in finding potential controls on this proxy. The first principal component (PC1) of seawater δ^18^O records (Fig. 3c) covaries with the strength of the AMOC (Fig. 3b). This finding suggests that inter-hemispheric thermal contrast controls rainfall-related changes in the salinity of the IPWP during abrupt climate events at millennial scale (Fig. 3a) [7]. The spatial pattern of the PC1 loadings indicates that sites in the central and western IPWP contribute most strongly to this component, whereas the easternmost ‘Pacific’ sites contribute little (Fig. S2c). The absence of an AMOC signal at these sites may reflect the influence of South Pacific water masses on the equatorial Pacific, as recently demonstrated [75]. The second principal component (PC2) of seawater δ^18^O (Fig. 3c) follows the SITIG, which is primarily controlled by precession (Fig. 3f) [46]. Nearly all sites across the IPWP exhibit substantial loadings on PC2, indicating that this component reflects a common, large-scale response to precessional forcing. In addition, this indicates that millennial events are superimposed on long-term, orbital-forced changes in ocean salinity and rainfall. In contrast, PC1 and PC2 of speleothem δ^18^O records (Fig. 3d) follow changes in sea level (Fig. 3g) and September insolation at the equator (Fig. 3f), respectively, suggesting that changes in moisture source and the state of ENSO dominate this signal. This interpretation is supported by uniformly positive PC1 loadings, consistent with a common sea-level signal, across all sites (Fig. S2a), while positive PC2 loadings are restricted to the equatorial sites (Fig. S2b). This interpretation is also consistent with site-specific interpretations of the speleothem data from Borneo [34,36] and Flores [51]. Lastly, PC1 and PC2 of the δD records (Fig. 3e) follow sea-level (Fig. 3g) and precession/SITIG changes (Fig. 3f), respectively. Similar to the speleothems, the PC1 of the δD records exhibits consistently positive loadings across all records (Fig. S2a). In contrast, the PC2 is dominated by the two Sulawesi records and should be treated with caution (Fig. S2b). A more robust assessment would require additional δD records from the eastern and southern IPWP to improve the spatial coverage of the dataset. Overall, these results highlight that different proxies might reflect different forcings of AIM rainfall in the past (see also Fig. S2).

**Figure 3.**
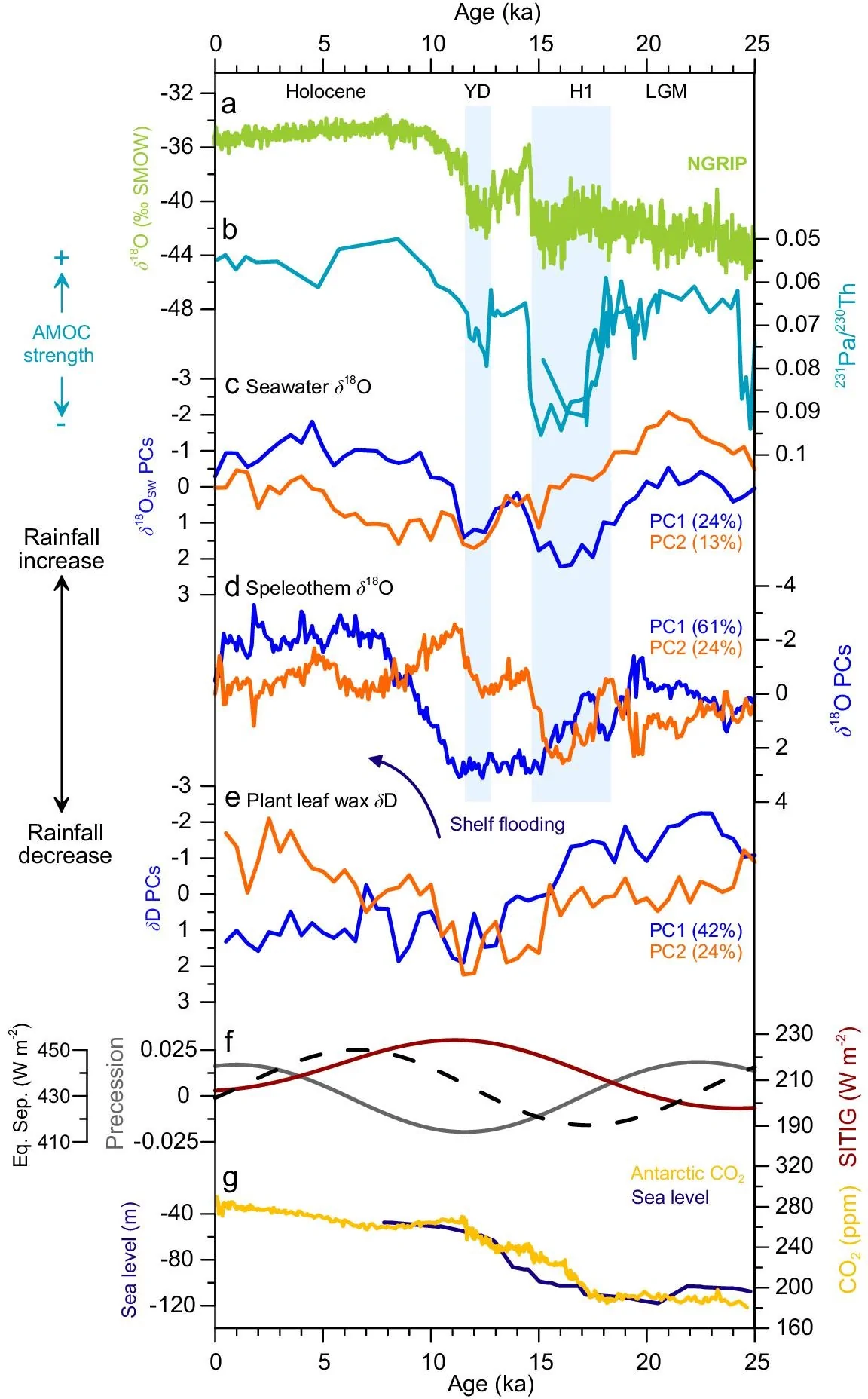
Millennial changes in isotope-based AIM proxy records. (a) δ^18^O data of Greenland ice core NGRIP [104,105] in per mil (‰) standard mean ocean water (SMOW). (b) ^231^ Pa/^230^Th records from the Bermuda Rise [106,107] as indicator for the strength of the AMOC. (c, d and e) 1st (blue) and 2nd (orange) principal components (PCs) of seawater δ^18^O (c), speleothem δ^18^O (d), and plant leaf wax δD (e) records. For site locations, see Fig. 1. (f) Precession index (gray), equatorial September insolation (dashed black) [103], and SITIG [46] (red, 21 June insolation 23°N–21 June insolation 23°S) in Watt per square meter (W m^−2^). (g) Antarctic composite ice core CO_2_ (yellow) [108] in part per million (ppm), and regional sea level (blue) [109] in meters (m) relative to today. Vertical blue bars indicate Heinrich Stadial 1 (H1) and the Younger Dryas (YD). LGM, Last Glacial Maximum.

Proxy records also indicate that the magnitude of the AIM responses to climate forcing at millennial scale depends strongly on orbital background state. The same Heinrich forcing can produce large rainfall anomalies during some intervals but weak or ambiguous responses during others, as documented in long speleothem sequences from Flores [71]. Precessional forcing controls the background latitude and seasonal intensity of the rainbelt, determining whether millennial scale perturbations can generate detectable rainfall anomalies at a given site. For instance, when Southern Hemisphere summer insolation is high, the rainbelt is already displaced southward, allowing millennial scale forcing to generate large rainfall responses at southern margin sites. On the contrary, when the rainbelt is positioned farther north, the same forcing may not affect rainfall to the same degree [76]. Millennial scale AIM variability is therefore state dependent and nonlinear [2,77].

### Multi-decadal to centennial oscillations: Indo-Pacific zonal gradients

Proxy records with a high temporal resolution provide an important bridge between paleoclimate and the modern system, capturing variability in zonal circulation, warm pool structure, and the expression of coupled climate phenomena, that is, ENSO and IOD, that also dominate interannual variability today. Speleothem data from Borneo suggest that periods of enhanced IPWP convection and rainfall, notably the mid-Holocene, coincide with reduced ENSO variance and a stronger Pacific Walker circulation [34,78]. Speleothem reconstructions over the last two millennia further highlight low-frequency variability in Pacific Walker circulation and reveal challenges for models, including underestimation of low-frequency variability in coupled simulations used for comparison [79]. High-resolution flood lamination records from northern Australia [80] and Java [81] show that besides changes in mean climate, multi-centennial variability in extreme rainfall from tropical cyclones is important to understand the impact of ENSO mean state and monsoon variability. Finally, coral records reveal temporal variability in IOD activity over the past several millennia and link Holocene background state to IOD event character [27]. Altogether, multi-decadal to centennial scale proxy records demonstrate that similar to today, AIM rainfall and wind have been strongly influenced by the mean state of the Walker circulation in the past.

### Reconciling robust conclusions from reconstructions

A critical interpretational consideration is that monsoon-sensitive archives should exhibit a clear bias toward recording monsoon-season conditions, and that model-proxy comparisons should therefore be seasonally explicit wherever possible. However, this assumption may not hold for some classical monsoon proxies, including speleothem and seawater δ^18^O records. Albeit this potential bias, robust conclusions emerging from the proxy synthesis are the following:

- AIM variability is intrinsically spatially heterogeneous today, and was so in the past.
- Orbital forcing, that is, precession and obliquity, controls AIM wind and rainfall by changing the interhemispheric tropical insolation gradient.
- Glacial weakening of rainfall in the interior Maritime Continent is primarily evaporation limited, driven by shelf exposure and stronger seasonality, while rainfall appears less affected at the Pacific and Indian Ocean margins of the Maritime Continent.
- Millennial-scale abrupt climate events reorganize the AIM via interhemispheric energy balance and changes in the position and strength of Hadley cell and ITCZ.
- The mean state of tropical convection and Walker circulation exerts a strong control on AIM rainfall, and is not limited to interannual timescales.

The synthesis above identifies a set of robust, process-level constraints on AIM behavior. We now evaluate to what extent current climate models reproduce these constraints, and where systematic discrepancies remain.

## MODELING THE AIM: PROCESSES, SKILLS AND LIMITATIONS

The proxy synthesis demonstrates that variability of the AIM arises from the interaction of multiple processes: interhemispheric energy balance, land-sea geometry, orbital preconditioning, moisture availability, and zonal coupling through the Walker circulation. Climate models are therefore required to reproduce not only changes in low-level circulation, but also the efficiency of moisture supply, the spatial organization of convection, and the nonlinear, state-dependent nature of monsoon rainfall. Model-proxy comparison thus provides a stringent test of model fidelity in one of the most dynamically complex regions of the climate system. However, it is noteworthy that even the last two generations of climate models in the Coupled Model Intercomparison Projects (CMIP) 5 and 6 continue to exhibit substantial shortcomings in simulating modern AIM dynamics with persistent biases in precipitation distribution, wind intensity, and seasonal transition, thus limiting their utility for impact assessments [4,82]. Notwithstanding, we focus mainly on results from these two generations of climate models, as results show that model performance strongly depends on its resolution in the AIM region [12].

For this synthesis, we additionally analyzed PMIP4-CMIP6 model simulations of the mid-Holocene (Fig. 4, 10 models: ACCESS-ESM1-5, AWI-ESM-1–1-LR, CESM2, EC-Earth3-LR, GISS-E2-1-G, HadGEM3-GC31-LL, INM-CM4-8, IPSL-CM6A-LR, MPI-ESM1-2-LR, and NorESM2-LM) and of the LGM (Fig. 5, three models: INM-CM4-8, MPI-ESM1-2-LR, AWI-ESM-1–1-LR). We assessed the ensemble-mean difference between the mid-Holocene and pre-industrial (PI) simulations, as well as between LGM and PI simulations. Inter-model uncertainty, that is, uncertainty arising from model disagreement, was quantified as the ensemble standard deviation of the 100-year mean MH–PI and LGM–PI differences (Figs 4 and 5).

**Figure 4.**
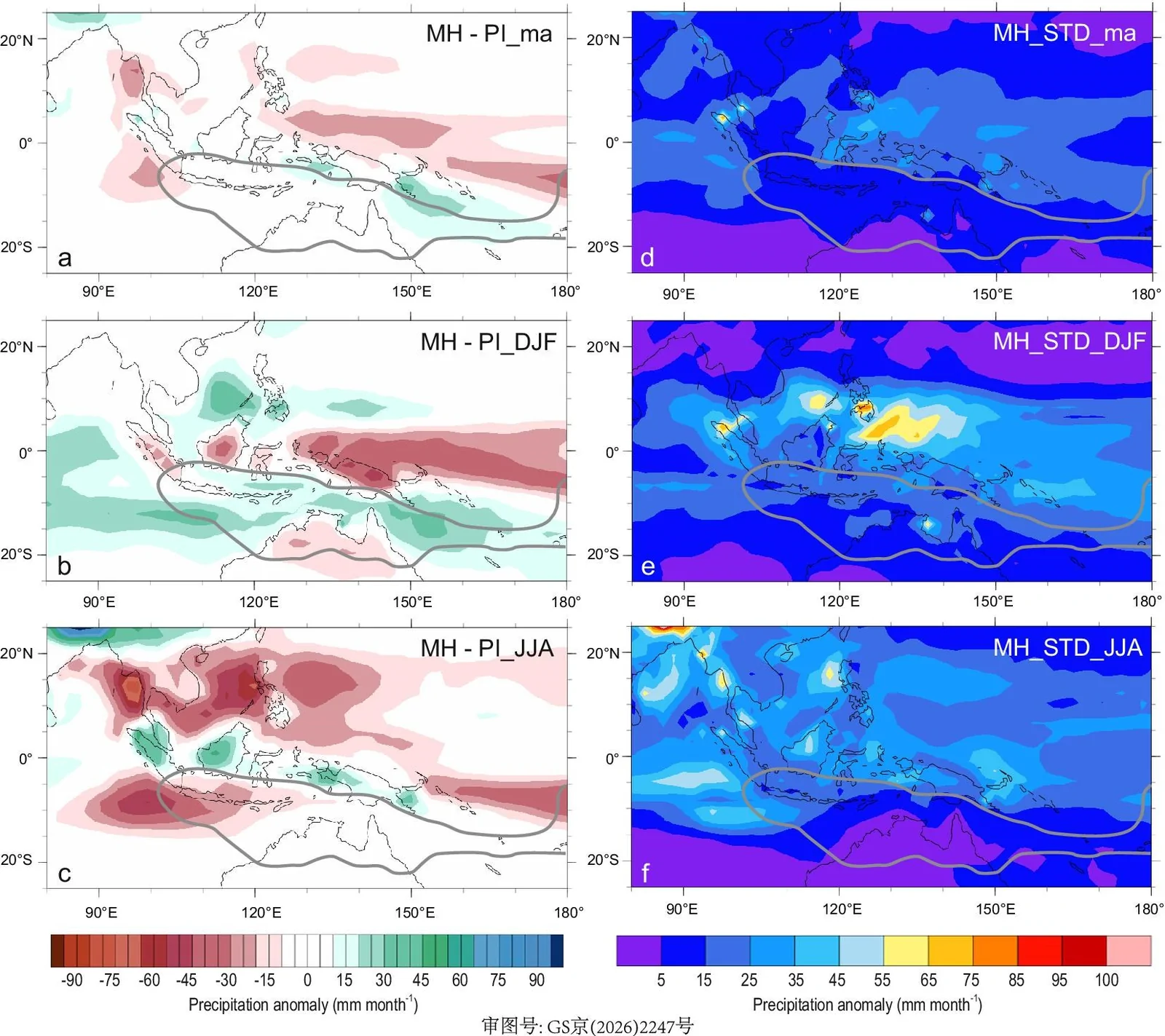
Ensemble mean precipitation anomalies (mm month^−1^) for the mid-Holocene relative to pre-industrial. (a–c) Simulated mid-Holocene (MH) minus pre-industrial (PI) precipitation in mm month^−1^ for (a) mean annual (ma), (b) the rainy season from December to February (DJF), and (c) the windy season from June to August (JJA). (d–f) Inter-model spread of the mid-Holocene climate response, expressed as the ensemble standard deviation of the 100-year mean MH–PI differences across the 10 PMIP4-CMIP6 models. The ensemble mean is based on 10 PMIP4-CMIP6 models (ACCESS-ESM1-5, AWI-ESM-1–1-LR, CESM2, EC-Earth3-LR, GISS-E2-1-G, HadGEM3-GC31-LL, INM-CM4-8, IPSL-CM6A-LR, MPI-ESM1-2-LR, and NorESM2-LM). Ensemble means and differences were averaged over the final 100 years of the simulations. Thick gray lines indicate the modern boundary of the AIM. Model outputs for all simulations were downloaded from the Earth System Grid Federation (ESGF; https://esgf-node.llnl.gov/).

**Figure 5.**
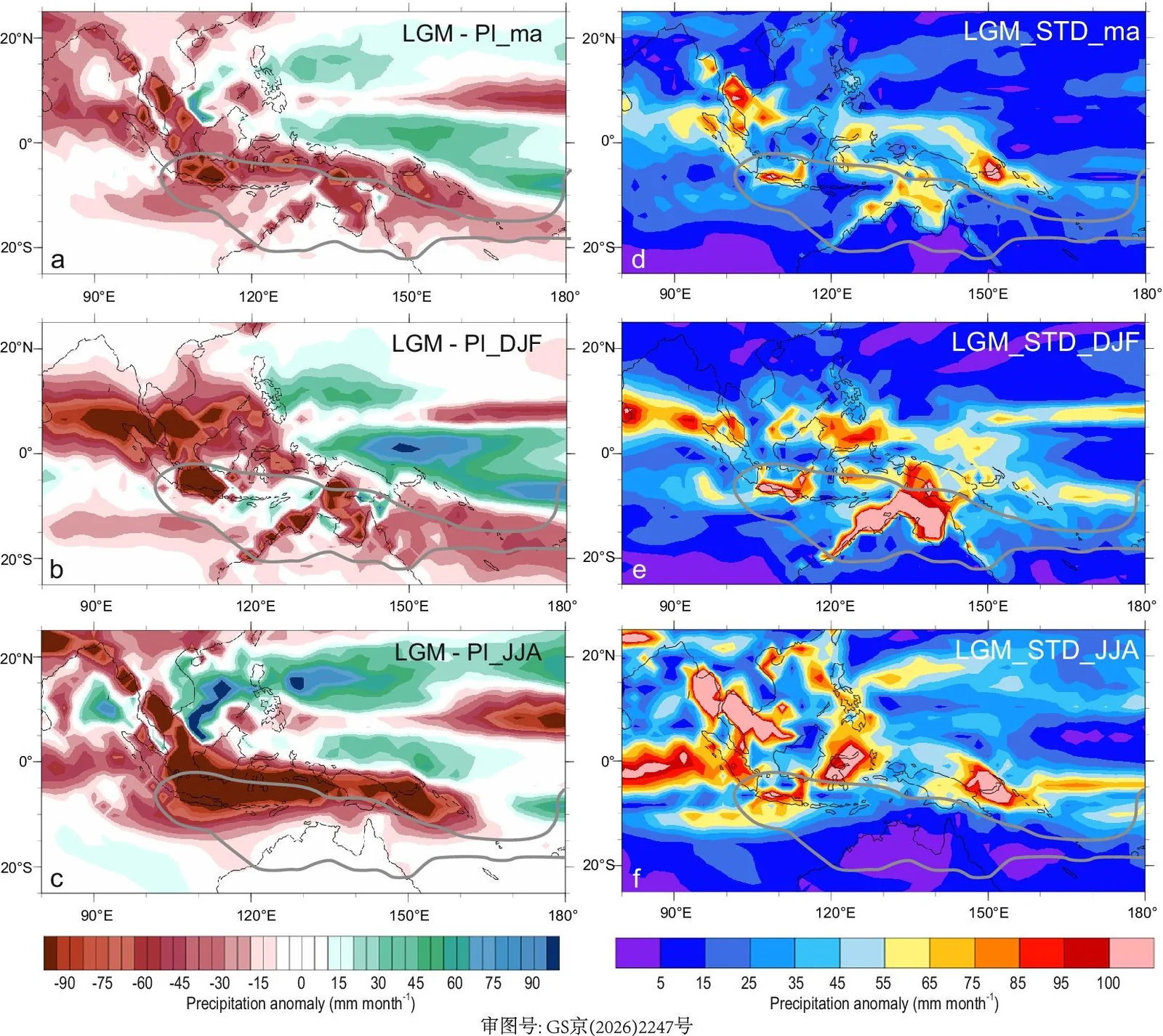
Ensemble mean precipitation anomalies (mm month^−1^) for the LGM relative to pre-industrial. (a–c) and (d–f) same as Fig. 4 but for the LGM using the three PMIP4-CMIP6 models INM-CM4-8, MPI-ESM1-2-LR, and AWI-ESM-1–1-LR. Model outputs for all simulations were downloaded from the Earth System Grid Federation (ESGF; https://esgf-node.llnl.gov/).

### Orbital forcing of monsoon circulation and rainfall

Climate models robustly simulate the response of monsoon circulation to insolation forcing. Experiments spanning single or multiple orbital cycles show that precession and obliquity modulate the distribution of insolation, altering the intensity and timing of austral-summer heating over northern Australia and SST in the Maritime Continent [2,83,84]. This leads to systematic changes in monsoon winds and low-level convergence that are broadly consistent with the orbital pacing observed in proxy records across the AIM realm. Earlier studies indicate that the Northern Hemisphere insolation has an important influence on the AIM strength, either by affecting the Siberian High and cross-equatorial winds [85], or through controls on the changing land and ocean boundary conditions over the northern Sahul [86].

Individual and ensemble mean of CMIP 5 and CMIP 6 model generations show AIM contraction and precipitation decrease over land during the mid-Holocene compared to the preindustrial period (Fig. 4b and c), mainly due to insolation changes [87] and a stronger Indian Walker circulation that is shifted westward with the main updraft over the ocean [88]. This scenario is broadly consistent with reconstructions from the core of the AIM realm—from Lesser Sunda Islands [31,51,89] and Timor Sea [65]—showing a drier mid-Holocene compared to late Holocene. However, the ensemble-mean simulation of AIM rainfall during the mid-Holocene suggests that the overall drying over the southern Maritime Continent was primarily driven by a drier dry season [87] (Fig. 4c), whereas over northern Australia, drying was mainly associated with reduced wet-season rainfall [87] (Fig. 4b). An earlier orbital experiment on the AIM rainfall indicates that increased insolation leads not only to a stronger land–sea thermal contrast but also an additional moisture inflow into the monsoon region of northwestern Australia from Southern Hemisphere midlatitudes [90]. This might explain relatively wetter mid-Holocene recorded in stalagmites from NW Australia [91].

Another simulation of the mid-Holocene suggests that September insolation maximum forces a warming in the IPWP thermocline and a stronger zonal thermal difference across the equatorial Pacific during the mid-Holocene, a scenario similar to La Niña conditions nowadays, and increased precipitation over the Maritime Continent [32]. This outcome is supported by data just north of the AIM realm from Borneo [34] and adjacent seas [32]—indicating wetter mid-Holocene compared to the late Holocene. However, this interpretation is challenged by the multi-model mean of the CMIP6 mid-Holocene simulations, which shows a negative precipitation anomaly over this region in both the annual mean and during the peak ENSO season (December–February; Fig. 4a and b). The CMIP6 multi-model mean further indicates that during the rainy season, the rainbelt was positioned between Australia and the equator (Fig. 4b), whereas during the dry season, it was located near the equator (Fig. 4c). Nevertheless, the inter-model spread in both seasonal and annual precipitation is nearly as large as the simulated anomalies themselves (Fig. 4d–f), suggesting that the simulated precipitation response to mid-Holocene orbital forcing is not robust across models.

### Interhemispheric energy balance and rainbelt reorganization

Coupled atmosphere–ocean general circulation models consistently reproduce the first-order response of tropical precipitation to interhemispheric energy balance perturbations. Freshwater hosing experiments and idealized simulations of the weakening of the AMOC produce cooling in the Northern Hemisphere, warming in the Southern Hemisphere, and a southward reorganization of the ITCZ [7,33,64,92]. In these simulations, rainfall decreases across the Northern Hemisphere tropics and increases in parts of the Southern Hemisphere tropics, broadly consistent with the proxy evidence from the northern and southern Maritime Continent during Heinrich stadials and the Younger Dryas mentioned above. Isotope-enabled transient climate model simulations implicate an additional zonal component in the Indian Ocean with a stronger Walker circulation over the Indian Ocean, akin to the modern negative IOD mode, during these periods [93].

Models also capture the sensitivity of the southern limit of austral summer convection to changes in hemispheric temperature gradients, reproducing enhanced austral monsoon circulation during Southern Hemisphere warming and suppressed convection during Northern Hemisphere warming [94,95]. These results provide a solid dynamical basis for interpreting millennial-scale AIM variability in terms of interhemispheric energy balance.

### The role of shelf exposure

Multi-model experiments indicate that during the LGM, mean annual precipitation decreased [96] while seasonality increased [82], without a significant shift of ITCZ position [7,82,95]. Rather, changes in AIM have been attributed to higher land-sea thermal contrast and the east-west SST gradients over the Indian and western Pacific oceans, both caused by an altered land-sea configuration due to sea level drop [82]. Simulations of the LGM further indicate the emergence of an equatorial mode of climate variability in the Indian Ocean, similar to the modern Pacific El Niño, as a response to exposure of the Sunda and Sahul shelves due to lower glacial sea level [97]. The exposure and inundation of Sunda and Sahul shelves appear critical in triggering the necessary feedback mechanisms [56], although they appear model-dependent [53]. An isotope-enabled transient climate experiment suggests that exposure of NW Australian shelf excited feedbacks across the equatorial Indian Ocean, and a weaker Walker circulation, while the influence of Sunda and Arafura shelves exposure excited a much weaker atmosphere-ocean response and resulted only in local drying [30]. Moreover, sensitivity experiments with different convective schemes and parametrizations suggest that thermal properties of an emerged Sunda Shelf increased moisture convergence and continental precipitation [19].

Ensemble mean results from CMIP6/PMIP4 indicate widespread drying, with the largest anomalies located over the exposed shelf regions [96] (Fig. 5a–c). However, similar to the mid-Holocene simulations, the model spread for the LGM is nearly as large as the rainfall anomalies (Fig. 5d–f), indicating considerable uncertainty in the simulated precipitation response to glacial boundary conditions. The above findings show that rainfall response to shelf emergence depends on the modeling frameworks, spatial resolution, parametrization, and convection scheme [19]. It should be noted that the shelf exposure during glacial lowstands occurs in association with decreased atmospheric CO_2_ and expanded polar ice-masses, diagnosing their respective effects may be one key to better resolve the AIM behavior during the LGM.

## KEY MISMATCHES IN PROXY-MODEL COMPARISON

The synthesis above reveals systematic discrepancies between modeled and reconstructed AIM behavior. These mismatches are not random; they point to specific missing or poorly represented processes.

1. Maritime Continent convection biases: Convection schemes frequently struggle to represent the observed sensitivity of rainfall to land-sea geometry, diurnal cycles, and shallow seas. As a consequence, simulated rainfall responses to shelf exposure and flooding are often spatially misplaced or of incorrect magnitude. For instance, all CMIP generations simulate a uniformly dry AIM domain during glacial lowstands and vice versa, indicating limits in the ability of models to reproduce the strong spatial heterogeneity documented by proxies across different regions during the LGM. Moreover, attribution of shelf effects remains difficult because sea level lowstands covary with low CO_2_ and large ice sheets. The most compelling model evidence therefore comes from sensitivity experiments in which sea level (land mask and bathymetry) is altered while CO_2_, ice sheets, and orbital parameters are held fixed [5,56]. Such experiments have established a ‘shelf fingerprint’ of rainfall, convergence, and moisture source change that is attributable to geography alone. However, two limitations remain unsolved. The first is paleogeographic uncertainty. Shelf bathymetry, shallow-sea geometry, and the representation of narrow straits matter for both atmospheric and oceanic responses in this region but are poorly represented in climate models, and also evolve through time due to shelf subsidence [19]. The second is model physics over the Maritime Continent. When nearly all models misrepresent land-sea rainfall distribution and convection in the modern climate, their shelf sensitivity may not be credible.
2. Underestimation of low-frequency Walker circulation variability: Proxy records reveal pronounced centennial-scale variability in the Walker circulation and its impact on AIM rainfall, particularly during the last two millennia [79]. In contrast, last millennium and historical simulations from CMIP ensembles typically concentrate variance at interannual to decadal timescales, with much weaker centennial-scale variability in zonal circulation and associated rainfall [98–100]. This deficiency is particularly evident when comparing reconstructed Medieval Climate Anomaly and Little Ice Age hydroclimate patterns with model simulations. Proxy records indicate prolonged periods of altered Walker circulation strength, whereas models tend to produce shorter-lived anomalies that are insufficient to account for the magnitude and persistence of observed rainfall changes [79].
3. Linear responses versus threshold behavior: Proxy evidence from equatorial IPWP sites demonstrates that deep tropical convection exhibits threshold behavior, responding strongly to Heinrich stadials but weakly or not at all to most Dansgaard–Oeschger events [36,67]. Many models, however, respond approximately linearly to freshwater forcing or insolation perturbations with insufficient representation of local to regional climate feedbacks [101].
4. Diagnosis of fine-scale spatial patterns by both proxy and model: The heterogeneity among reconstructions carries an important conceptual implication for the AIM. Mechanisms such as shelf exposure require proxy networks that resolve gradients and contrasts rather than only regional averages. However, while disagreements among proxy records can reflect genuine spatial structure, apparent agreement among proxies may reflect shared sensitivity to moisture source or seasonality rather than to precipitation amount. The goal is therefore not only to compile more records, but to strengthen inference by integrating multi-proxy networks with proxy-specific modeling that can distinguish among amount, seasonality, and transport changes.

## IMPLICATIONS FOR FUTURE MODELING STRATEGIES

The proxy-model comparison highlights several priorities for improving simulations of the AIM:

1. Explicit representation of shelf geometry and shallow seas, including their effects on evaporation and boundary-layer energy balance. Regional models can better resolve coastal and topographic processes that are central to Maritime Continent rainfall organization, though they are less commonly deployed for long integrations and require careful boundary conditions.
2. Improved convection schemes for the Maritime Continent that capture land–sea contrasts, diurnal cycles, and convective thresholds. This is particularly important for the AIM domain as exposure and flooding of the Sunda and Sahul shelves changes land fraction, shifts coastlines, and modifies the distribution of shallow seas across the region most central to the monsoon’s convective core.
3. Isotope-enabled transient simulations spanning orbital to millennial timescales, to disentangle rainfall amount from moisture-source effects. They also provide a valuable bridge to speleothem and marine isotope proxies, allowing model-data comparisons in isotopic space rather than relying solely on precipitation fields.
4. Long, continuous simulations capable of generating centennial-scale Walker circulation variability comparable to proxy reconstructions, ideally as an ensemble approach to help identify where responses are robust across models.

Addressing these challenges is essential not only for understanding past monsoon variability, but also for improving confidence in projections of future hydroclimate change in one of the world’s most densely populated and climatically sensitive regions.

## SUMMARY

The Australian–Indonesian monsoon is exceptionally sensitive to continental evolution because its convective core lies over the Maritime Continent, where coastlines, shallow seas and topography organize rainfall. Quaternary sea level change, through exposure and flooding of the Sunda and Sahul shelves can shift the position and seasonality of convection, not merely its intensity. CO_2_, ice sheets, and orbital insolation impose additional forcing, frequently transmitted through Indo-Pacific SST pattern changes and largescale circulation adjustments. Most defensible mechanistic understanding comes from Quaternary studies, where proxy constraints and model experiments can be meaningfully compared. The next phase of progress will require routine ensembles, explicit quantification of paleogeographic uncertainty, improved simulation of Maritime Continent convection and the diurnal cycle, and proxy-aware evaluation using isotope-enabled modeling and multi-proxy approaches.

## Supplementary Material

nwag473_Supplemental_Files

## Data Availability

Model output for the Last Glacial Maximum, mid-Holocene, and pre-industrial simulations of all three models are available at the Earth System Grid Federation (ESGF, https://esgf-node.llnl.gov/). References and sources of the original publications are listed in Table S1.
