# Supplementary material for: The Australian–Indonesian monsoon through time
Source: Natl Sci Rev. 2026 Aug 5;13(17):nwag473. doi: 10.1093/nsr/nwag473 (PMC13639189; doi:10.1093/nsr/nwag473)
Supplement: Supplementary file 1 [file nwag473_supplemental_files.zip › Figure S1_Mohtadi et al.pdf]

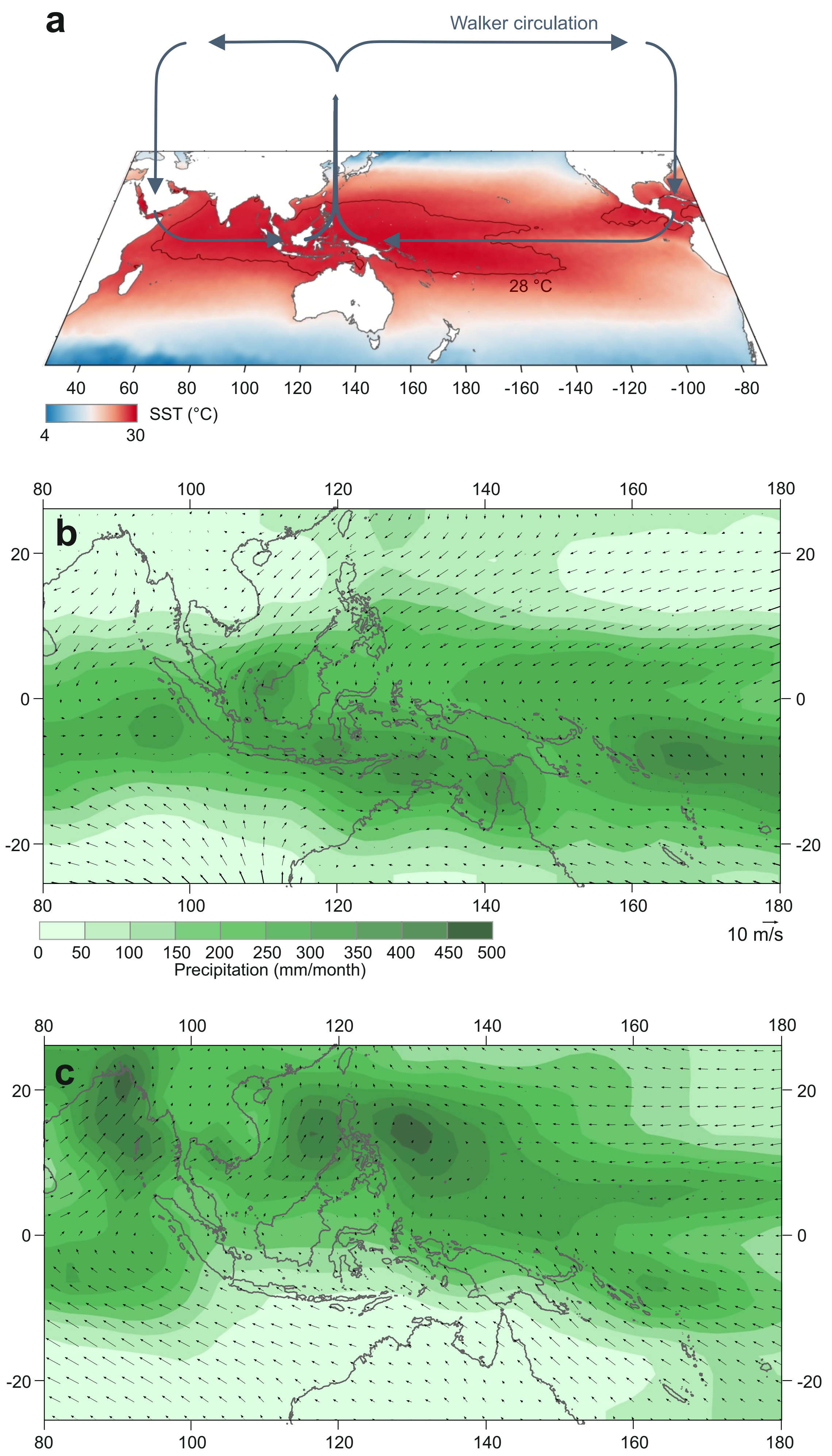
a
Walker circulation
28 °C
40 60 80 100 120 140 160 180 -160 -140 -120 -100 -80
SST (°C)
4
30
b
80 100 120 140 160 180
20 0 -20
0 50 100 150 200 250 300 350 400 450 500
Precipitation (mm/month)
10 m/s
c
80 100 120 140 160 180
20 0 -20
