# Supplementary material for: The Australian–Indonesian monsoon through time
Source: Natl Sci Rev. 2026 Aug 5;13(17):nwag473. doi: 10.1093/nsr/nwag473 (PMC13639189; doi:10.1093/nsr/nwag473)
Supplement: Supplementary file 1 [file nwag473_supplemental_files.zip › Figure S2_Mohtadi et al.pdf]

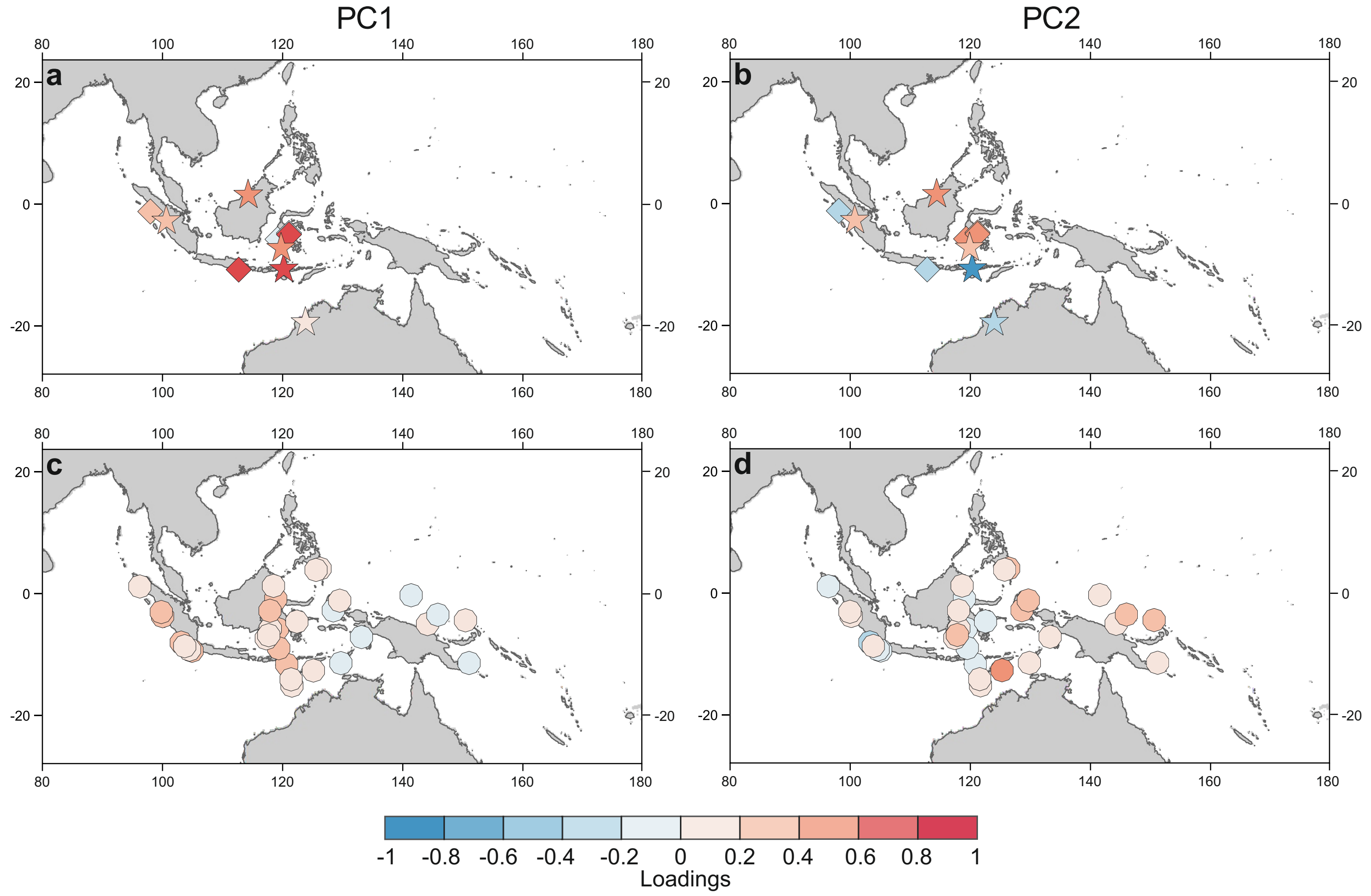

PC1
PC2
a
b
c
d
80
100
120
140
160
180
20
0
-20
-1
-0.8
-0.6
-0.4
-0.2
0
0.2
0.4
0.6
0.8
1
Loadings
