# Supplementary material for: The Australian–Indonesian monsoon through time
Source: Natl Sci Rev. 2026 Aug 5;13(17):nwag473. doi: 10.1093/nsr/nwag473 (PMC13639189; doi:10.1093/nsr/nwag473)
Supplement: Supplementary file 1 [file nwag473_supplemental_files.zip › Suppl. Table1_Mohtadi_NSR.docx]

| **Site** | **Latitude °N** | **Longitude °E** | **Reference** | **Reference doi** | **Data doi** |
| --- | --- | --- | --- | --- | --- |
| **Seawater δ^18^O** |  |  |  |  |  |
| GeoB10038-4 | -05.94 | 103.25 | Mohtadi et al.. 2010 | 10.1016/j.epsl.2010.01.024 | 10.1594/PANGAEA.770343 |
| GeoB10029-4 | -01.50 | 100.13 | Mohtadi et al.. 2010 | 10.1016/j.epsl.2010.01.024 | 10.1594/PANGAEA.736647 |
| 3cBx | 08.02 | 139.64 | Sagawa et al.. 2012 | 10.1016/j.palaeo.2012.06.002 | 10.1594/PANGAEA.965815 |
| GeoB10042-1 | -07.11 | 104.64 | Setiawan et al.. 2015 | 10.1002/2015PA002802 | 10.1594/PANGAEA.858594 |
| GeoB10043-3 | -07.31 | 105.06 | Setiawan et al.. 2015 | 10.1002/2015PA002802 | 10.1594/PANGAEA.858594 |
| GeoB10069-3 | -09.59 | 120.92 | Gibbons et al.. 2014 | 10.1016/j.epsl.2013.11.032 | 10.1594/PANGAEA.839404 |
| GeoB17426-3 | -02.19 | 150.86 | Hollstein et al.. 2020 | 10.1029/2019PA003832 | 10.1594/PANGAEA.910129 |
| GIK18471-1 | -09.37 | 129.98 | Capelli et al.. 2016 | 10.1016/j.palaeo.2016.09.010 | 10.1594/PANGAEA.864731 |
| GiK18515-3 | -03.63 | 119.36 | Schröder et al.. 2016 | 10.1016/j.quascirev.2016.10.018 | 10.1594/PANGAEA.868615 |
| GIK18519-2 | -00.57 | 118.11 | Schröder et al.. 2018 | 10.1029/2018PA003323 | 10.1594/PANGAEA.894078 |
| GIK18522-3 | 01.40 | 119.08 | Schröder et al.. 2018 | 10.1029/2018PA003323 | 10.1594/PANGAEA.894079 |
| GIK18526-3 | -03.61 | 118.17 | Schröder et al.. 2018 | 10.1029/2018PA003323 | 10.1594/PANGAEA.894080 |
| GIK18540-3 | -06.87 | 119.58 | Schröder et al.. 2018 | 10.1029/2018PA003323 | 10.1594/PANGAEA.894081 |
| KX973-21-2 | -01.42 | 157.98 | Dang et al.. 2020 | 10.1126/sciadv.abc0402 | 10.1594/PANGAEA.921277 10.1594/PANGAEA.922658 |
| KX973-22-4 | -00.03 | 159.25 | Zhang et al. 2017; 2021 | 10.1007/s00343-017-6082-9 10.1038/s43247-021-00305-5 | 10.1594/PANGAEA.939377 |
| MD01-2378 | -13.08 | 121.79 | Holbourn et al.. 2005 Xu et al. 2008 Dürkop et al.. 2008 Zuraida et al.. 2009 | 10.1029/2004PA001094 10.1016/j.epsl.2008.06.029 10.1016/j.marmicro.2007.10.002 10.1029/2008PA001653 | 10.1594/PANGAEA.263757 10.1594/PANGAEA.831197 10.1594/PANGAEA.666553 10.1594/PANGAEA.712513 |
| MD01-2386 | 01.13 | 129.79 | Jian et al.. 2020 | 10.1073/pnas.1915510117 | 10.1073/pnas.1915510117 |
| MD05-2925 | -09.34 | 151.46 | Lo et al.. 2022 | 10.1016/j.quascirev.2022.107756 | 10.1594/PANGAEA.952143 10.1594/PANGAEA.952137 |
| MD06-3067 | 06.51 | 126.50 | Bolliet et al.. 2011 | 10.1029/2010PA001966 | 10.1594/PANGAEA.770282 |
| MD10-3340 | -0.52 | 128.72 | Dang et al.. 2020 Jian et al.. 2022 | 10.1126/sciadv.abc0402 10.1038/s41586-022-05302-y | 10.1594/PANGAEA.967044 10.1594/PANGAEA.921277 10.1594/PANGAEA.950059 |
| MD97-2138 | -01.25 | 146.23 | de Garidel-Thoron et al.. 2007 | 10.1029/2006PA001269 | 10.25921/dhkp-bs81 |
| MD97-2140 | 02.04 | 141.76 | de Garidel-Thoron et al.. 2005 | 10.1038/nature03189 | 10.25921/ejer-t729 |
| MD97-2141 | 08.79 | 141.30 | Oppo et al.. 2003 Dannenmann et al.. 2003 Rosenthal et al.. 2003 | 10.1029/2001GC000260 10.1029/2002GC000390 10.1029/2002GL016612 | https://www.ncei.noaa.gov/pub/  data/paleo/paleocean/sediment_files/ |
| MD98-2161 | -05.22 | 117.49 | Fan et al.. 2018 | 10.1038/s41598-018-24055-1 | 10.25921/hwh4-xh41 |
| MD98-2162 | -04.69 | 117.90 | Jian et al.. 2022 | 10.1038/s41586-022-05302-y | 10.1594/PANGAEA.950060 |
| MD98-2170 | -10.59 | 125.39 | Stott et al.. 2007 | 10.1126/science.114379 | 10.25921/5rmm-9t86 |
| MD98-2176 | -05.00 | 133.44 | Stott et al.. 2007 | 10.1126/science.114379 | 10.25921/5rmm-9t86 |
| MD98-2178 | 03.62 | 118.70 | Fan et al.. 2018 | 10.1038/s41598-018-24055-1 | 10.25921/hwh4-xh41 |
| MD98-2181 | 06.30 | 125.83 | Stott et al.. 2002 | 10.1126/science.1071627 | 10.25921/9sj9-hj52 |
| SO139-74KL | -06.54 | 103.83 | Lückge et al.. 2009 Wang et al.. 2018 | 10.1029/2008PA001627 10.1016/j.quascirev.2018.05.035 | 10.1594/PANGAEA.882099 10.1594/PANGAEA.894990 |
| SO189-39KL | -00.79 | 99.91 | Mohtadi et al.. 2014 | 10.1038/nature13196 | 10.1594/PANGAEA.971792 |
| SO18480 | -12.06 | 121.65 | Jian et al.. 2022 | 10.1038/s41586-022-05302-y | 10.1594/PANGAEA.950061 |
| TGS-931 | -02.41 | 122.62 | Schröder et al.. 2018 | 10.1029/2018PA003323 | 10.1594/PANGAEA.894082 |
| GeoB17419 MD05-2920 | -02.81 | 144.50 | Tachikawa et al.. 2014 Hollstein et al.. 2018 | 10.1016/j.quascirev.2013.12.018 10.1016/j.quascirev.2018.10.030 | 10.1594/PANGAEA.965889 10.1594/PANGAEA.899082 |
| SO189-119KL | 03.52 | 96.31 | Mohtadi et al.. 2014 | 10.1038/nature13196 | 10.1594/PANGAEA.963956 |
|  |  |  |  |  |  |
| **Leaf wax δD** |  |  |  |  |  |
| Lake Towuti | 121.30 | -02.75 | Konecky et al.. 2016 | 10.1016/j.epsl.2015.12.037 | 10.25921/qqd9-q059 |
| Lake Matano | 121.30 | -02.50 | Wicaksono et al.. 2015 Parish et al.. 2024 | 10.1016/j.quascirev.2024.108755 10.1016/j.palaeo.2015.04.016 | 10.25921/gjsk-jn37 |
| SO189-144KL | 98.07 | 01.16 | Niedermeyer et al.. 2014 | 10.1073/pnas.1323585111 | 10.1594/PANGAEA.836162 |
| BJ8-70GGC | 119.38 | -03.57 | Tierney et al.. 2012 | 10.1029/2012JD018060 | 10.25921/s8sh-1059 |
| GeoB10053-7 | 112.87 | -08.68 | Ruan et al.. 2018; 2025 | 10.1016/j.quascirev.2019.06.015 10.1029/2025GL114695 | 10.1594/PANGAEA.896146 |
| MD98-2152 | 103.88 | -06.33 | Windler et al.. 2020 | 10.1029/2020PA003985 | 10.1594/PANGAEA.920834 |
|  |  |  |  |  |  |
| **Speleothem δ^18^O** |  |  |  |  |  |
| Gunung Buda | 114.48 | 04.02 | Partin et al.. 2007 Carolin et al.. 2013; 2016 | 10.1038/nature06164 10.1126/science.123379 10.1016/j.epsl.2016.01.028 | 10.25921/rxkn-e268 |
| Liang Luar Cave | 120.44 | -08.54 | Griffiths et al.. 2009 Lewis et al.. 2011 Ayliffe et al.. 2013 | 10.1038/ngeo605 10.1016/j.epsl.2010.12.048 10.1038/ncomms3908 | 10.25921/m36w-f623 |
| Tangga Cave | 100.76 | -00.36 | Wurtzel et al.. 2018 | 10.1016/j.epsl.2018.04.001 | 10.25921/ft28-dh46 |
| Ball Gown Cave | 124.08 | -17.33 | Denniston et al.. 2013 | 10.1016/j.quascirev.2013.04.012 | 10.25921/0558-fw77 |
| Gempa bumi cave | 120.00 | -05.00 | Krause et al.. 2019 | 10.1016/j.epsl.2019.01.045 | 10.25921/am5m-z254 |
