# Supplementary material for: The Australian–Indonesian monsoon through time
Source: Natl Sci Rev. 2026 Aug 5;13(17):nwag473. doi: 10.1093/nsr/nwag473 (PMC13639189; doi:10.1093/nsr/nwag473)
Supplement: Supplementary file 1 [file nwag473_supplemental_files.zip › supplementary information.docx]

**Fig S1. Modern setting of the Australian-Indonesian Monsoon.**

**(a)** Annual mean SST of the World Ocean Atlas 2023 (*110*) with a schematic illustration of the Walker circulation over the equatorial Pacific and Indian oceans. The contour line of 28°C marks the boundary of the IPWP. **(b-c)** Precipitation amount in mm/month (green shadings) with average wind speed and directions at 1000 hPa (black arrows, note the scale at the bottom right corner of the panels) across the AIM domain during austral summer in January (**b**) and austral winter in July (**c**). SST and precipitation data are averaged between 1991-2020 and provided by the U.S. National Centers for Environmental Prediction (NCEP), maps are modified from iridl.ldeo.columbia.edu.

**Fig S2. Spatial pattern of the first and second Principal Components (PC1 and PC2) of the isotope-based proxy records.**

**(a-b)** Loading of speleothem δ^18^O (stars) and δD (diamonds) records. **(c-d)** Loading of seawater δ^18^O records.

**Table S1. Sites shown in Fig. 1 and included in the principal component analysis, with DOI links to the corresponding references and data sources.**
